# Reverse-D-4F Increases the Number of Endothelial Progenitor Cells and Improves Endothelial Progenitor Cell Dysfunctions in High Fat Diet Mice

**DOI:** 10.1371/journal.pone.0138832

**Published:** 2015-09-23

**Authors:** Yang Nana, Jiao Peng, Zhang Jianlin, Zhang Xiangjian, Yao Shutong, Zhan Enxin, Li Bin, Zong Chuanlong, Tian Hua, Si Yanhong, Du Yunsai, Qin Shucun, Wang Hui

**Affiliations:** 1 College of Animal Science and Technology, Shandong Agricultural University, Tai'an, China; 2 Key Laboratory of Atherosclerosis in Universities of Shandong, Institute of Atherosclerosis, Taishan Medical University, Taian, China; 3 Hebei Collaborative Innovation Center for Cardio-cerebrovascular Disease and Hebei Key Laboratory of Vascular Homeostasis, Shijiazhuang, China; 4 Central Hospital of Taian, Taian, China; University of Kansas Medical Center, UNITED STATES

## Abstract

Although high density lipoprotein (HDL) improves the functions of endothelial progenitor cells (EPCs), the effect of HDL ApoAI mimetic peptide reverse-D-4F (Rev-D4F) on EPC mobilization and repair of EPC dysfunctions remains to be studied. In this study, we investigated the effects of Rev-D4F on peripheral blood cell subpopulations in C57 mice treated with a high fat diet and the mechanism of Rev-D4F in improving the function of EPCs impaired by tumor necrosis factor-α (TNF-α). The high fat diet significantly decreased the number of EPCs, EPC migratory functions, and the percentage of lymphocytes in the white blood cells. However, it significantly increased the number of white blood cells, the percentage of monocytes in the white blood cells, and the level of vascular endothelial growth factor (VEGF) and TNF-α in the plasma. Rev-D4F clearly inhibited the effect of the high fat diet on the quantification of peripheral blood cell subpopulations and cytokine levels, and increased stromal cell derived factor 1α (SDF-1α) in the plasma. We provided in vitro evidence that TNF-α impaired EPC proliferation, migration, and tube formation through inactive AKT and eNOS, which was restored by Rev-D4F treatment. In contrast, both the PI3-kinase (PI3K) inhibitor (LY294002) and AKT inhibitor (perifosine) obviously inhibited the restoration of Rev-4F on EPCs impaired by TNF-α. Our results suggested that Rev-D4F increases the quantity of endothelial progenitor cells through increasing the SDF-1α levels and decreasing the TNF-α level of peripheral blood in high fat diet-induced C57BL/6J mice, and restores TNF-α induced dysfunctions of EPCs partly through stimulating the PI3K/AKT signal pathway.

## Introduction

Diet has been shown to play an important role in the development of cardiovascular disease, which remains the major cause of death in western countries [1]. The balance between endothelial injury and repair is a key component of atherosclerosis [2]. Both neighboring endothelial cells and endothelial progenitor cells (EPCs) from bone marrow and peripheral tissues participate in repairing endothelial injury induced by risk factors including diabetes, hypercholesterolemia, hypertension, and smoking [3, 4, 5, 6]. It has been shown that a high fat diet contributes to the impairment of vascular function both in healthy subjects and in patients with cardiovascular disease (CVD) [7], as well as the reduction of the circulating levels of EPCs after critical limb ischemia [8] and the increase of the level of leukocytes in the peripheral blood [9]. Based on those observations, we speculated that a high fat diet induced peripheral blood micro environment changes (such as changes in the level of total cholesterol [TC], nitric oxide [NO], stromal cell derived factor 1α [SDF-1α], vascular endothelial growth factor [VEGF], tumor necrosis factor [TNF]-α, etc.), indirectly influencing endothelial repair through mobilization of bone marrow cell and peripheral blood progenitor cell transdifferentiation.

ApoAI mimetic peptide D4F (with 4 phenylalanine residues) synthesized with D-amino acids has been demonstrated to reduce atherosclerosis in apolipoprotein E (apoE)-null and LDL-receptor-null mice [10, 11]. Reverse-D-4F (Rev-D4F), a modified D4F with reverse order, can also decrease aortic sinus atherosclerotic lesion area and lesion macrophage content by inhibiting endothelial inflammatory/oxidative events and improving the high density lipoprotein (HDL) function [12]. Recently, we found that Rev-D4F improved mice bone marrow-derived EPC functions through the PI3K/AKT/eNOS pathway [13]. However, the effect of Rev-D4F on peripheral blood cell populations in C57BL/6J mice treated with high fat diets remains unclear. Therefore, in this study, we assessed the effect of Rev-D4F on the number of different cell populations in the peripheral blood such as EPCs, lymphocytes, neutrophils, and monocytes, the inflammation of the arterial wall induced by a high fat diet, and the improvement of EPC functions impaired by the inflammatory factor TNF-α through the PI3K/AKT pathway.

## Materials and Methods

### Mimetic peptide synthesis

The D-4F peptide had the following amino acid sequence: Ac-DWFKAFYDKVAEKFKEAF-NH2. The Reverse D4F mimetic peptide was synthesized by the Scilight-Peptide INC (Beijing, China) using approaches of inverse chirality with D-amino acids only and reverse order, respectively. The structure and purity (98%) were determined by GC/MS.

### Animals

6-week-old C57BL/6J mice were purchased from the Vital River Company (Beijing, China) and randomly divided into four groups. Four groups containing 6 mice each were fed either a normal chow diet, a normal chow diet with Rev-D4F (1mg/kg/d), a high fat diet (15.8% fat and 1.25% cholesterol), or a high fat diet with Rev-D4F (1mg/kg/d) for 16 weeks. All animal procedures conformed to the Guide for the Care and Use of Laboratory Animals published by the Taishan Medical University, and the protocol was approved by the Committee on the Ethics of Animal Experiments of Taishan Medical University. All surgeries were performed under chloral hydrate anesthesia.

### Flow cytometric quantification of endothelial progenitor cells

Peripheral blood derived from the C57BL/6J mice was treated with red blood cell-lysing buffer (BD), and labeled with the FITC-conjugated antibodies against CD34 (BD) and PE- conjugated antibodies against FLK-1 (BD). Debris and nonspecific fluorescent signals were excluded by employing a gating strategy and isotype-identical antibodies.

### Immunofluorescent (IF) staining

Serial aortic root cryosections were stained with c-kit (CST), sca-1 (Abcam), ICAM-1 (Santa Cruz), and VCAM-1 (Santa Cruz). Images were captured using a fluorescent microscope (× 40).

### C57BL/6J mice bone marrow-derived EPCs isolation

The mice were sacrificed by dislocation after anesthesia with chloral hydrate. Mice bone marrow-derived mononuclear cells (MNCs) were isolated by density gradient centrifugation with Ficoll as previously described [14]. MNCs were seeded on fibronectin-coated plates at the density of 10^6^/cm^2^ in EBM-2MV (Lonza, plus SingleQuots^TM^ of growth supplements). After four days, non-adherent cells were removed with PBS, and fresh medium was added to the cultures every three days.

### EPCs functions analysis

EPCs were pretreated with the PI3K inhibitor LY294002 (30μM) for 2 h, and were incubated with Rev-D4F (50μg/ml) for 6 h. After TNF-α was added to the medium for 24 h, EPCs were digested with 0.25% trypsin (Sigma) to investigate the functions of EPCs on proliferation, migration, and tube formation in vitro as previously described [14].

EPCs were incubated with DiI-ac-LDL (Molecular Probes, 2.5 mg/L) for 2 h at 37°C, and FITC-UEA (Sigma, 10 mg/L) for 1 h at 37°C after fixed for 5 min with 1–2% paraformaldehyde. These staining cells were randomly selected five fields of view and counted double positive cells numbers and total cell numbers under a fluorescence microscope (× 10). The positive cells (%) were number of double positive cells compared with the total number of cells.

EPCs proliferation was determined by MTT. Different groups of cells were incubated with 20μl MTT (5mg/ml, Sigma) for 4 h at 37°C. Each well was added with 150 μL dimethyl sulfoxide (DMSO, Gibco) and shaked for 10 min. OD value was measured at 490nm.

EPCs migration was measured by using a 8μm pore 24-well Cell Migration Assay kit (BD). 1.2×10^4^ cells in M199 were added to the upper chamber and EGM-2MV medium was added to the lower compartment. After 24 h incubation at 37°C, removed the upper cells with a cotton wool swab, fixed and stained transwell filters with DAPI. The migratory cells were counted in five random microscopic fields (× 10).

Cells were digested with 0.25% trypsin, and 1.0×10^4^ cells were added to matrigel in 96 wells plate. After 18 h incubation, the average of the total length of complete tubes was detected under a microscope (×4) in five random microscopic fields by using computer software, Image-Pro Plus.

### Western blot analysis

Total protein was extracted with RIPA lysis buffer, and was quantified by the BCA method. 30 μg protein was electrophoresed on a 8% denaturing polyacrylamide gel, transferred onto PVDF membranes, and blocked with 5% dried skimmed milk. The membranes were incubated with antibodies against β-actin (1:20000, Sigma), AKT (1:2000, Cell Signaling), phosphor-AKT (1:1000, Cell Signaling), eNOS (1:500, Santa Cruz), phosphor-eNOS (1:1000, Cell Signaling), iNOS (1:500, Santa Cruz) for 2 h, and subsequently incubated with horseradish peroxidase-conjugated goat anti-rabbit IgG antibody (1:3000, Santa Cruz). The signals were detected using the Phototope-HRP Western Detection Kit (Thermo).

### Plasma lipid analysis

Total plasma cholesterol was measured by enzymatic method (BioSino) according to the manufacturer's protocol.

### Plasma VEGF, SDF-1α, TNF-α and NO measurement

The plasma concentration of VEGF, SDF-1α, and TNF-α were determined by an ELISA kit (Blue Gene) according to the manufacturer's instructions.

The level of NO in plasma was measured by using the method of nitrate reductase, with a NO assay kit (Nanjing Jiancheng Institute of Biological Engineering, China) following the manufacturer’s instructions.

### Statistical analysis

All data are presented as mean ± SD. Differences between groups were assessed by one-way ANOVA using the software program SPSS11.5. All correlations between the levels of plasma cell factors and the numbers of EPCs were determined with spearman product–moment estimates. Values of P<0.05 were considered to be significant.

## Results

### The effect of Rev-D4F on the level of TC, TG, NO, VEGF, SDF-1α and TNF-α in blood plasma

**Fig 1:**
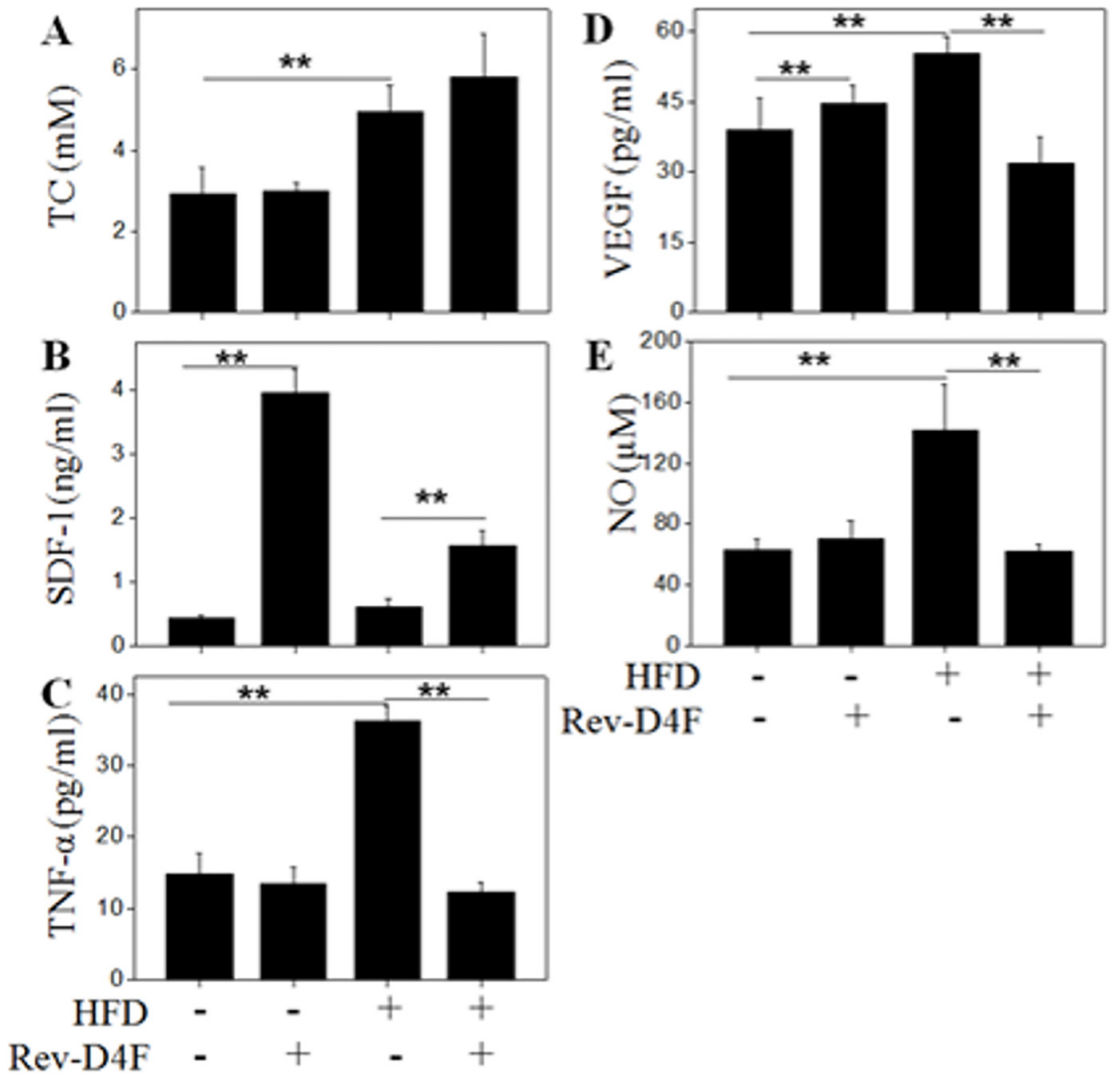
The effect of Rev-D4F on plasma lipid level, NO, and cytokines (VEGF, TNF-α and SDF-1α) in different groups of mice. A, The level of total cholesterol was significantly increased in the high fat diet group of mice. B, C, and D show different groups of mice blood cytokine (SDF-1α, TNF-α and VEGF) levels. E, The blood NO level in different groups of mice was measured by an NO assay kit at 550nm. Data are presented as mean±SD (n = 6). **P <0.01.

Plasma TC in mice was significantly increased after 16 weeks treatment with a high fat diet (Fig 1A). Rev-D4F did not significantly change plasma TC in mice fed either the chow diet or high fat diet. The increased levels of TNF-α, VEGF and NO (Fig 1C–1E) in mice plasma induced by the high fat diet were significantly reduced in the Rev-D4F treatment group. In addition, plasma SDF-1α (Fig 1B) was significantly increased in the Rev-D4F treatment group compared with those in the high fat diet or chow diet groups (Fig 1B).

### The effect of Rev-D4F on EPC number and function in vivo

**Fig 2:**
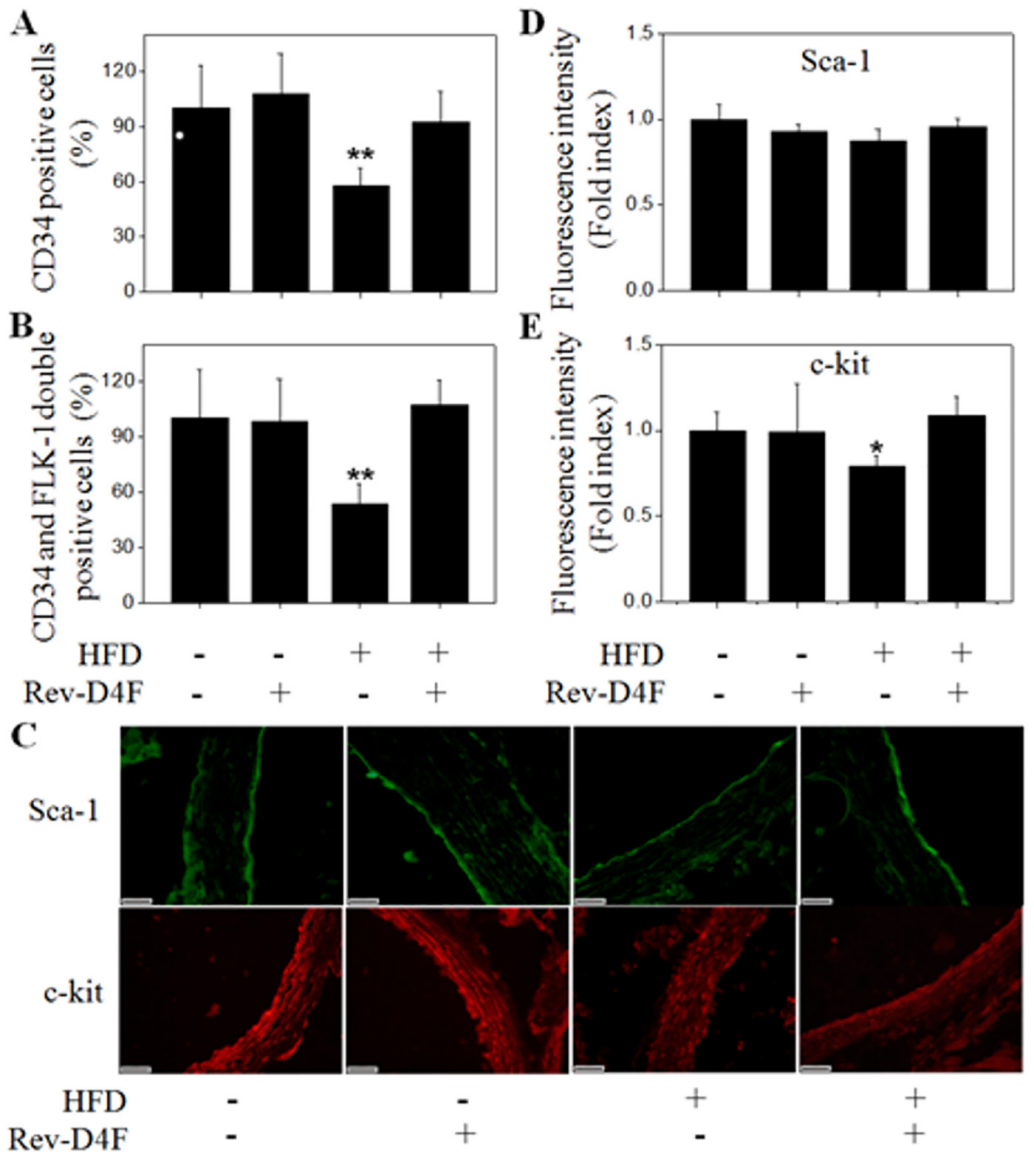
EPCs were detected in the peripheral blood and arterial walls derived from the different groups of mice. Numbers of CD34^+^ cells (A) and CD34^+^/FLK-1^+^ cells (B) from mice peripheral blood in percentage of control. **C**, Green fluorescence represented sca-1 positive cells in mice arterial walls, and red fluorescence represented c-kit positive cells in mice arterial walls. Fluorescence intensity was calculated for the expression of sca-1 (D) and c-kit (E). Relative fluorescence intensity in percentage of control. Scale bar represented 20μm. Data are presented as mean±SD (n = 6). *P <0.05, **P <0.01 versus chow diet group.

The circulating level of EPCs (CD34, CD34/FLK-1) was significantly lower in the high fat diet group compared with the chow diet group. However, Rev-D4F improved the high fat diet-induced EPC decrease in C57BL/6J mice peripheral blood (Fig 2A and 2B). Analysis of thoracic aortic sections also showed that the high fat diet significantly decreased the number of EPCs (c-kit^+^) (Fig 2C and 2E), whereas Rev-D4F obviously increased the number of c-kit^+^ EPCs to restore impaired aortic endothelium induced by the high fat diet (Fig 2E).

**Fig 3:**
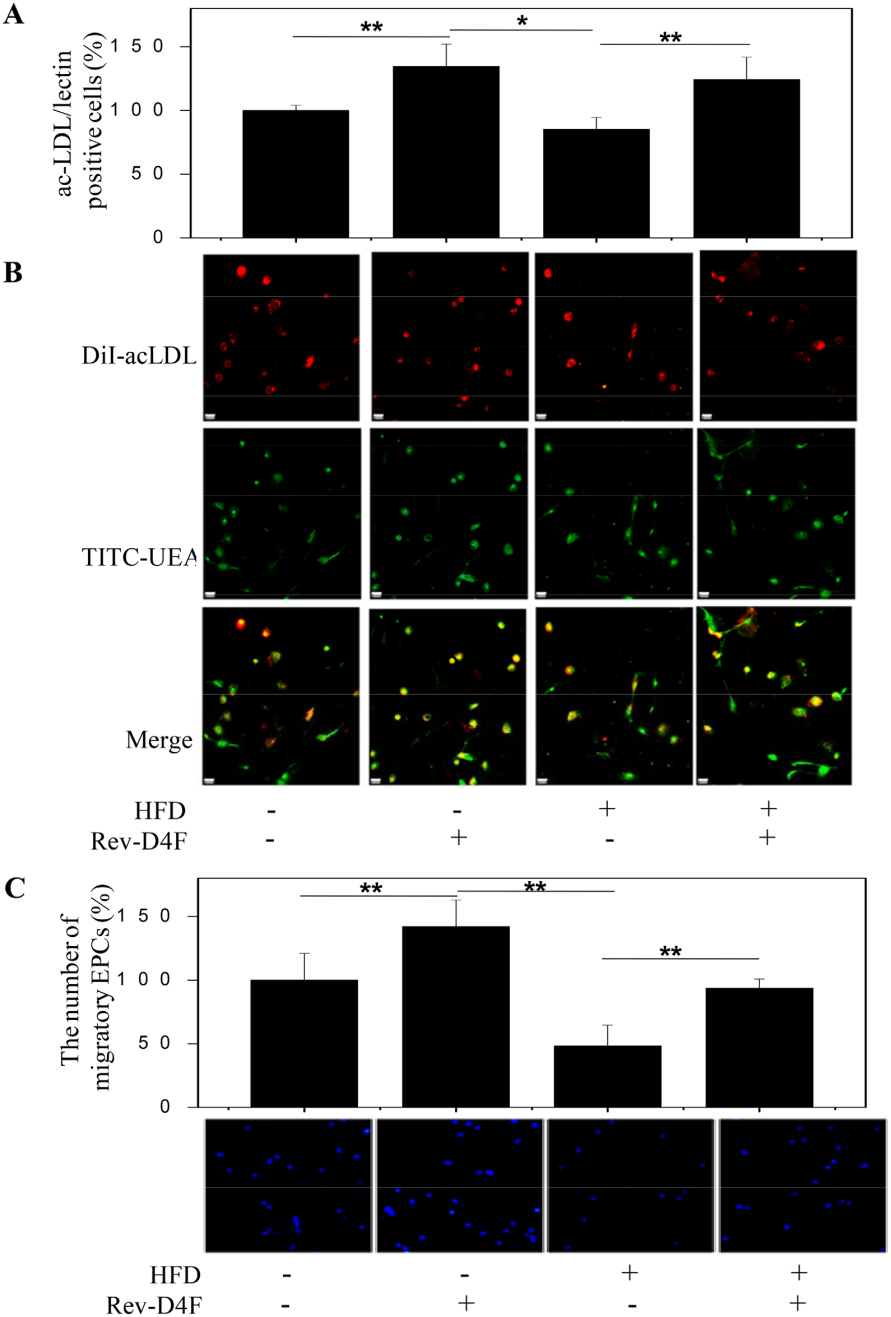
EPCs number and migratory functions in different groups of mice. A, EPCs numbers in different groups of mice. Numbers per high-power field in percentage of control. B, Different groups of mice mononuclear cells were isolated and cultured for 10 days to detect DiI-acLDL/lectin double-positive cells. Merge: green lectin, red Dil-acLDL. C, Mononuclear cells were isolated and cultured for 10 days to detect the migratory functions of EPCs using the transwell method. Numbers per high-power field in percentage of control. Scale bar represented 20μm. Data are presented as mean±SD (n = 6). **P <0.01.

EPCs derived from all groups were seeded on fibronectin-coated plates in EGM-2MV medium and cultured for 10 days to measure DiI-acLDL/lectin double-positive cells and migratory function. Results showed that the high fat diet led to a decrease in DiI-acLDL/lectin double-positive cell number (Fig 3A and 3B) and migration (Fig 3C). However, Rev-D4F restored the number of DiI-acLDL/lectin double-positive cells (Fig 3A and 3B) as well as the migratory function impaired by the high fat diet (Fig 3C).

### Rev-D4F improved the number of lymphocytes, and decreased the number of leukocytes, neutrophils, and monocytes in the peripheral blood of high fat diet-induced C57 mice

**Fig 4:**
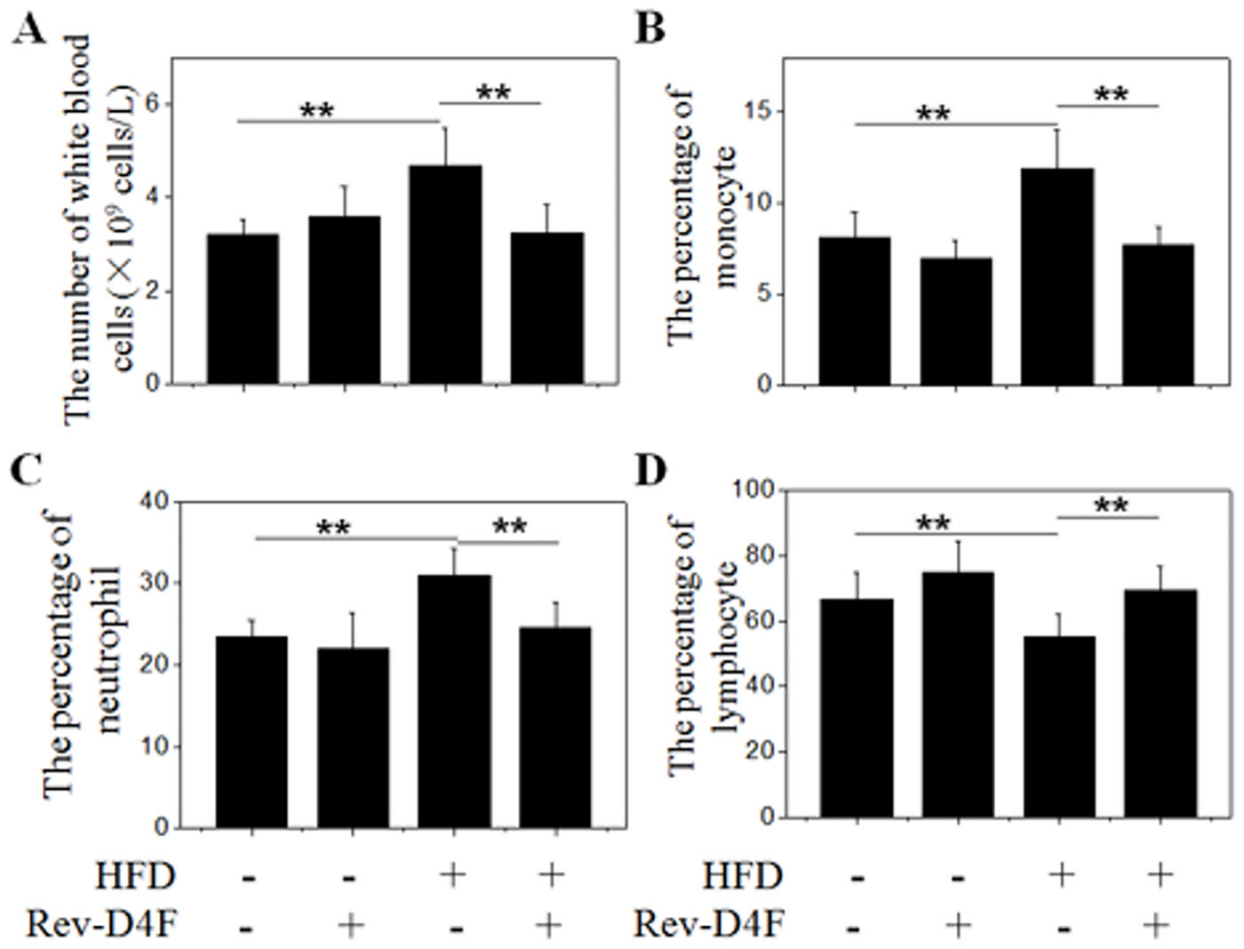
The proportion of different leukocyte subsets in mice blood. A, Representative of the number of white blood cells per liter. B, C, and D Monocytes, neutrophils, and lymphocytes in percentage of white blood cells, respectively. Data are presented as mean±SD (n = 6). **P <0.01.

Further analysis of the C57 mice peripheral blood indicated that Rev-D4F significantly decreased high fat diet-induced leukocytosis (Fig 4A). Among these leukocytes, the number of monocytes and neutrophils obviously increased in the high fat diet mice group compared with the chow diet group. Analysis of the peripheral blood in mice treated with Rev-D4F demonstrated that Rev-D4F obviously inhibited the high fat diet-induced increase in the numbers of leukocyte, neutrophils, and monocytes (Fig 4A–4C). In contrast, Rev-D4F significantly inhibited the peripheral blood lymphocyte reduction induced by the high fat diet (Fig 4D).

### Rev-D4F inhibited the expression of ICAM-1 and VCAM-1 in the arteries of high fat diet-induced C57 mice

**Fig 5:**
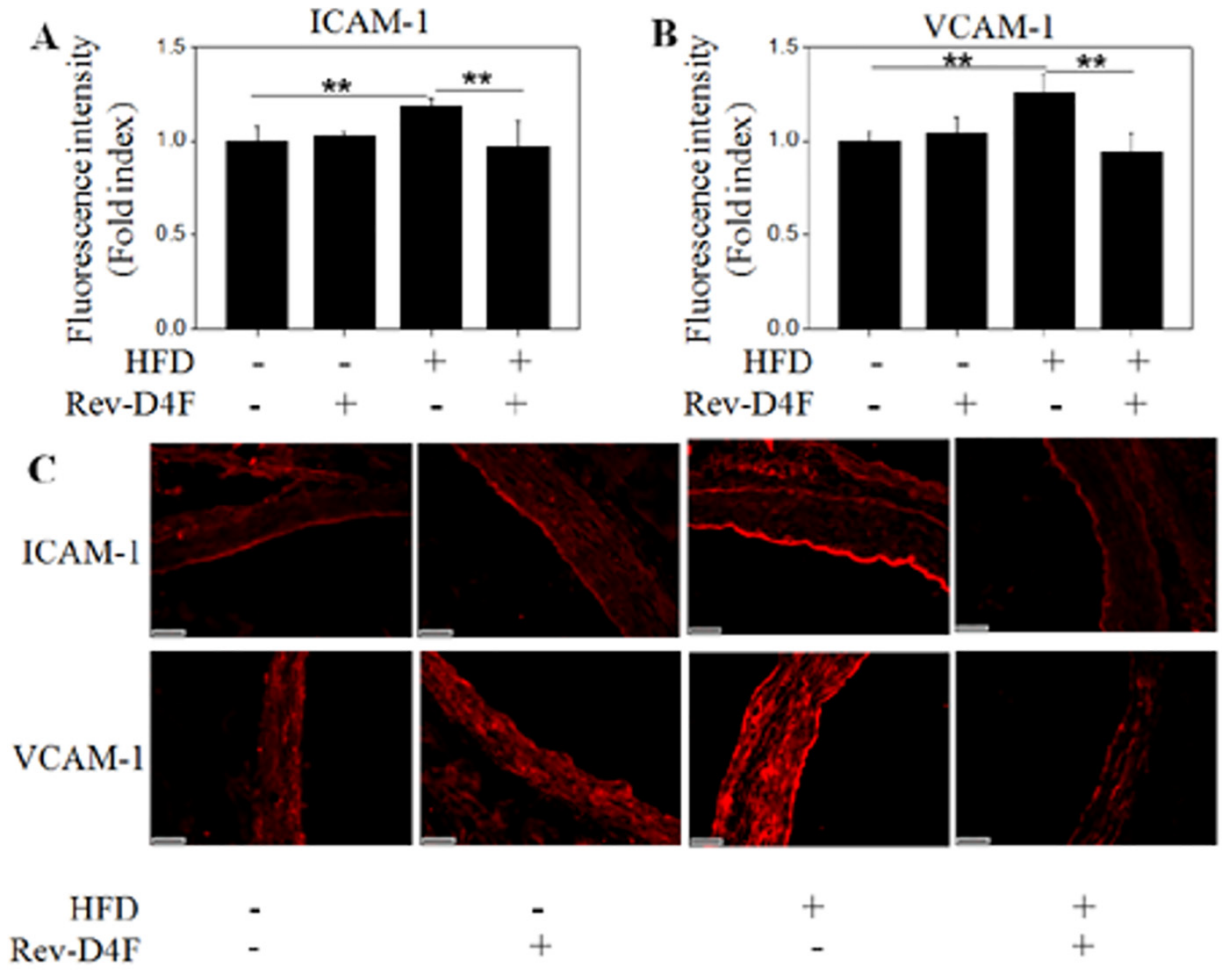
Rev-D4F decreased the expression of ICAM-1 and VCAM-1 in the arterial walls of C57BL/6J mice fed a high fat diet. Fluorescence intensity was calculated for the expression of ICAM-1 (A) and VCAM-1 (B). C, Representative of immunostained aortic sections with ICAM-1 and VCAM-1 antibodies. Relative fluorescence intensity in percentage of control. Scale bar represented 20μm. Data are presented as mean±SD (n = 6). **P <0.01.

The frequency of coronary artery disease was inversely associated with the level of circulating EPCs [15]. The major risk factors of atherosclerosis significantly improved the level of ICAM-1 or VCAM-1 expression in vascular intima [16]. In this study, immunofluorescence analysis of thoracic aortic sections showed that the expression of ICAM-1 and VCAM-1 was significantly decreased in mice that were fed a high fed diet with Rev-D4F, compared with the high fed diet group (Fig 5A–5C).

### Correlations between SDF-1α and TNF-α with EPC

**Fig 6:**
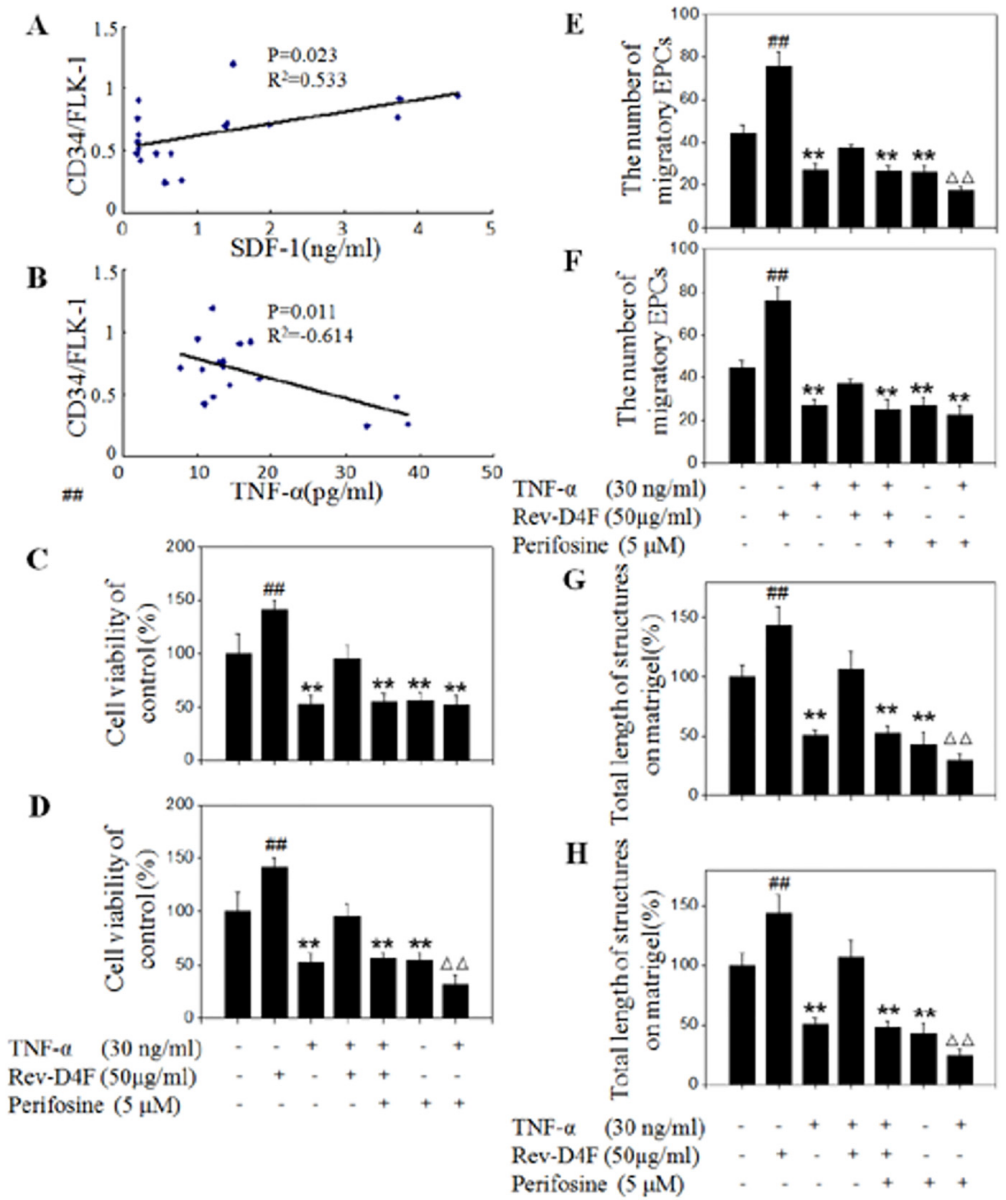
Correlations between SDF-1α and TNF-α with EPCs subsets in different groups of mice, and Rev-D4F inhibited TNF-α-induced impairment of EPCs functions. A, The increased level of SDF-1α significantly correlated with the increased number of the CD34/FLK-1 subset. B, TNF-α levels inversely correlated with CD34/FLK-1 subset numbers. Samples were pretreated with a PI3K inhibitor LY294002 (30μM) or AKT inhibitor perifosine (5μM) for 2 h and incubated with Rev-D4F (50μg/ml) for 6h, EPCs were treated with TNF-α for 24h to detect the functions of viability (C and D), migration (E and F) and tube formation (G and H). EPCs tube formation ability was calculated by the average of complete tube lengths. Data are means ± SD from at least three independent experiments. **P <0.01 versus control, ^*##*^ *P* <0.01 versus TNF-α, ^ΔΔ^ *P* <0.01 versus LY294002.

SDF-1α was demonstrated to be correlated with an increase in EPC during the early stage of ischemic stroke [17]. Rev-D4F significantly improved the level of SDF-1α in plasma both in the chow diet mice and high fat diet groups (Fig 1B). Further correlation analysis showed that SDF-1α levels moderately correlated with the EPC subset (CD34/FLK-1; r = 0.533; P = 0.023) (Fig 6A). However, TNF-α levels inversely correlated with the EPC (CD34/FLK-1) subset (r = -0.614; P = 0.011) (Fig 6B).

### Rev-D4F inhibited TNF-α-induced impairment of EPC functions

The above findings revealed that Rev-D4F inhibited the high fat diet-induced TNF-α level in plasma and improved the number of EPCs in the peripheral blood of the high fat diet-induced C57 mice. Therefore, we further investigated the potential effect of TNF-α and Rev-D4F on the functions of EPCs. In vitro experiments demonstrated that TNF-α impaired the proliferation of EPCs in a dose dependent manner, and it (≥30ng/ml) significantly inhibited EPC proliferation (S1A Fig). Other EPC functions, such as migration and tube formation, were also impaired by TNF-α (30ng/ml) (Fig 6C–6H). However, Rev-D4F inhibited the effect of TNF-α on the functions of EPC proliferation, migration, and tube formation (Fig 6C–6H).

### LY294002 inhibited the restoration of Rev-D4F on EPCs impaired by TNF-α

To further assess the protective effect of Rev-D4F on TNF-α-induced EPC impairment through the PI3K/AKT pathway, the functions of EPCs were detected when EPCs were pretreated with a PI3K inhibitor (LY294002) and AKT inhibitor (perifosine). LY294002 and perifosine significantly inhibited the repairing effect of Rev-D4F on EPC proliferation (Fig 6C and 6D), migration (Fig 6E and 6F), and tube formation (Fig 6G and 6H), that was impaired by TNF-α.

### Rev-D4F restored TNF-α-inhibited AKT and eNOS stimulation

**Fig 7:**
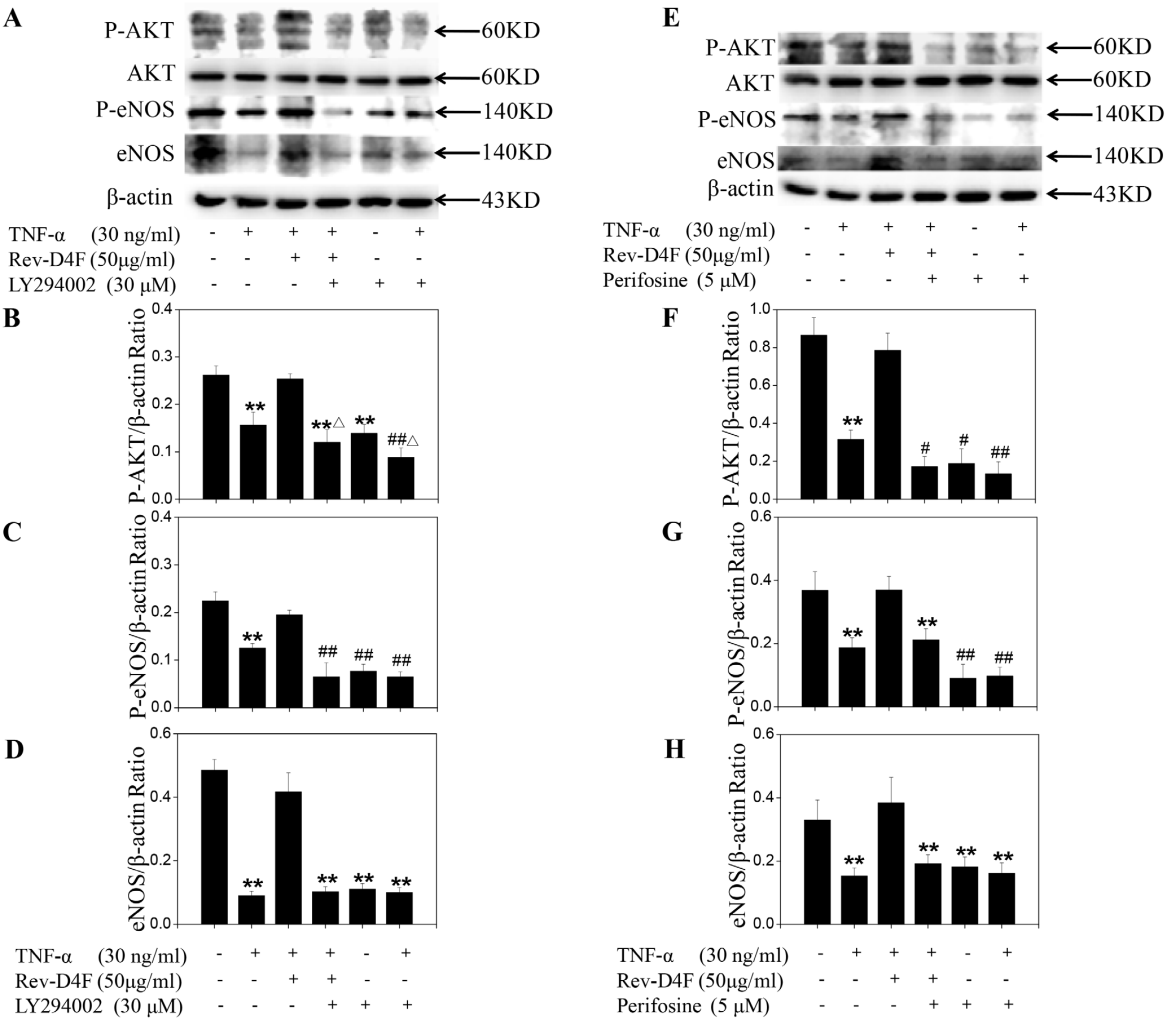
Activation effects of Rev-D4F on AKT and eNOS phosphorylation, and eNOS expression, inhibited by TNF-α. A, Western blot analysis of phosphor-AKT, eNOS, and phosphor-eNOS in EPCs treated with TNF-α, Rev-D4F, LY294002 or Perifosine. The density ratio of phosphor-AKT (B and F), phosphor-eNOS (C and G) and eNOS (D and H) to β-actin. Data are means ± SD from at least three independent experiments. **P <0.01 versus control, ^*#*^ *P* <0.05, ^*##*^ *P* <0.01 versus TNF-α, ^Δ^ *P* <0.05, ^ΔΔ^ *P* <0.01 versus LY294002.

Rev-D4F improved the functions of EPCs through activation of the AKT/eNOS signal pathway. AKT and eNOS phosphorylation and eNOS expression level were significantly reduced in EPCs treated with TNF-α. In our study, TNF-α (30ng/ml) significantly inhibited the expression level of eNOS (Fig 7A and 7E), as well as the phosphorylation of AKT (Fig 7A and 7E) and eNOS (Fig 7A and 7E). Pretreating EPCs with Rev-D4F for 6h significantly inhibited the TNF-α-induced eNOS downregulation (Fig 7A and 7D) and the decreased phosphorylation of AKT (Fig 7A and 7B) and eNOS (Fig 7A and 7C). Further studies were performed to show the association of AKT with eNOS. Western blotting analysis showed that pretreatment of EPCs with the PI3K inhibitor LY294002 and AKT inhibitor perifosine could obviously inhibit the phosphorylation of AKT and eNOS, as well as the expression level of eNOS (Fig 7A–7H). These results suggested that AKT phosphorylation contributed to eNOS expression and phosphorylation.

## Discussion

The differentiation of progenitor cells and stem cells is induced by microniches in the peripheral blood and bone marrow that include cytokines, inflammatory factors, plasma cholesterol, and lipid composition; these microniches also influence the occurrence and development of atherosclerosis [18, 19, 20, 21]. A high fat diet has been shown to decrease the number of EPCs and impair the functions of EPCs in obese rats [22]. It was also demonstrated that HDL could enhance EPC-mediated endothelium repair in mice [23], and that mimetic peptide D-4F could improve EPC functions and contribute to vascular repair in diabetic rats [24]. However little is known about the effect of a high fat diet on the number of EPCs and white blood cells in normal C57BL/6J mice, or about the effect of mimetic peptide Rev-D4F on progenitor cells mobilization and differentiation. Our results demonstrated that Rev-D4F significantly inhibited a high fat diet-induced decrease in EPCs (CD34, CD34/FLK-1) and lymphocytes in the C57BL/6J mice, and was associated with an increase in the number of progenitor cells (Sca-1, C-kit) homing to artery walls to repair the impaired endothelium. Rev-D4F was also associated with suppression of the high fat diet-induced increase of leukocytes, neutrophils, and monocytes in C57BL/6J mice.

To investigate the effect of Rev-D4F and high fat diet on normal animals, we used the C57BL/6J mouse as a model and divided them into four groups. The groups were fed a normal chow diet, a normal chow diet with Rev-D4F (1mg/kg/d), a high fat diet (15.8% fat and 1.25% cholesterol), or a high fat diet with Rev-D4F (1mg/kg/d). Our results showed that the level of TC was significantly increased in the plasma of mice fed a high fat diet, whereas Rev-D4F did not have a significant effect on TCs in the normal chow diet and high fat diet groups. These results were consistent with our previous report, and there was no difference in the levels of cholesterol between the control- and Rev-D4F-treated mice [12]. Analysis of results found that the numbers of EPCs and leukocytes (neutrophils, lymphocytes, and monocytes) were not related to the level of TCs in mice plasma in the different groups. Rossi and colleagues reported that, at normal HDL-C levels, there was no difference in early-EPC number either in hypercholesterolemic or nonhypercholesterolemic obese women [25]. We have also demonstrated that Rev-D4F had no effect on total plasma or HDL-cholesterol levels in apoE-null mice [12]. Therefore, changes of the numbers of EPCs and leukocytes in Rev-D4F-treated mice are not associated with the level of TCs in mice plasma due to unchanged HDL-cholesterol levels in plasma.

A high fat diet has been shown to augment the level of serum TNF-α in mice [26]. The level of serum TNF-α in congestive heart failure patients was inversely correlated with the number of EPCs in peripheral blood [27]. However, the apoA-I mimetic peptide L-4F significantly lowered the overexpression of TNF-α in db/db mice serum [28]. In this study, we find that Rev-D4F decreased the level of plasma TNF-α and increased the number of EPCs in the high fat diet-treated mice. In addition, the increase of TNF-α level induced by a high fat diet was closely associated with a reduction of EPCs (CD34, CD34/FLK-1). Apart from TNF-α, the number of EPCs (CD34, CD34/FLK-1) may also be associated with other factors, such as SDF-1α, VEGF, and NO [29, 30, 31]. EPCs pretreated with SDF-1α can increase their capacity to adhere to the activated endothelium, differentiate, and contribute to cell therapy for ischemic vascular diseases [32]. Little is known with respect to the effect of Rev-D4F on the level of SDF-1α in plasma. Our study showed that the level of plasma SDF-1α was significantly increased by Rev-D4F, and was not influenced by a high fat diet. Further analysis of the data of SDF-1α and EPCs indicated that there was obvious association between the level of SDF-1α and the number of EPCs. At present, VEGF and NO have been reported to contribute to EPC mobilization and proliferation, as well as improvement of EPC functions [32]. In our study, VEGF and NO, mainly as proinflammatory cytokines, were increased by a high fat diet, and were not correlated with the number of EPCs. Further study of the effects of EPCs on endothelium repair demonstrated that the expression levels of sca-1 and c-kit, also known as murine progenitor cells [33], were significantly decreased in high fat diet-treated mice thoracic aortic sections. Moreover, Rev-D4F obviously inhibited the decreased expression of c-kit induced by a high fat diet. Overall, the above results demonstrated that Rev-D4F could improve a high fat diet-induced decrease in the number of EPCs, which may be associated with the decrease in the level of TNF-α and the increase in the level of SDF-1α induced by Rev-D4F.

TNF-α is an independent risk factor for CHD [34], which can be used to predict the stability of coronary atherosclerotic plaques and the severity of acute coronary syndrome [35]. It is also involved in the development of atherosclerosis through promoting the adhesion of monocytes to vascular damaged endothelial cells [36]. Recently, TNF-α was identified to increase EPC apoptosis and damage EPC functions through decreasing the eNOS activity [37]. In the present study, we also found that the EPC functions, such as proliferation, migration, and tube formation, were impaired by TNF-α, which obviously inhibited AKT and eNOS phosphorylation, and eNOS expression. However, Rev-D4F inhibited the effect of TNF-α on the functions of EPC proliferation, migration, and tube formation, and inhibited the TNF-α-induced eNOS downregulation and decreased phosphorylation of AKT and eNOS. To clarify the role of the PI3K/AKT pathway in regulating EPC functions and eNOS phosphorylation and expression, EPCs were pretreated with LY294002 or perifosine for 2h and then treated with Rev-D4F and TNF-α. Both LY294002 and perifosine obviously inhibited the repaired effect of Rev-D4F on EPC functions damaged by TNF-α, and it inhibited eNOS phosphorylation and expression levels promoted by Rev-D4F. All these findings suggested that Rev-D4F improved the dysfunctions of EPCs induced by TNF-α, partially through the PI3K/AKT/eNOS signaling pathway.

## Conclusion

Rev-D-4F contributes to the increase in quantity of EPCs and improvement their dysfunctions in high fat diet-induced C57BL/6J mice, which may be associated with the level of TNF-α and SDF-1α in mice plasma. Rev-D-4F restored TNF-α-induced dysfunctions of EPCs, in part, by stimulating the PI3K/AKT signaling pathway. These results provide new insights into the role of lipoprotein ApoAI mimetic peptide for the treatment of atherosclerosis.

## Supporting Information

S1 Fig The effect of TNF-αand LY294002 on cell viability. A, The effect of different concentration of TNF-α on EPCs viability. **P <0.01 versus TNF-α (0 ng/ml). B, LY294002 inhibited the effect of Rev-D4F on EPCs viability. Samples were pretreated with different concentrations of a PI3K inhibitor LY294002 for 2 h and incubated with Rev-D4F (50μg/ml) for 6h, EPCs were treated with TNF-α for 24h to detect the functions of viability. **P <0.01.
(TIF)

S2 Fig Flow cytometric analysis of EPCs. Quantification of CD34^+^ and CD34^+^FLK-1^+^ were shown in S2 Fig. Control represents isotype-identical antibodies.
(TIF)

S3 Fig The effect of Rev-D4F on the repairmen of EPCs impaired by TNF-α. legend. Cells were pretreated with a PI3K inhibitor LY294002 (30μM) or AKT inhibitor perifosine (5μM) for 2 h and incubated with Rev-D4F (50μg/ml) for 6h, EPCs were treated with TNF-α for 24h to detect tube formation. Scale bar represented 50μm.
(TIF)
