# Supplementary figures and images for: Reverse-D-4F Increases the Number of Endothelial Progenitor Cells and Improves Endothelial Progenitor Cell Dysfunctions in High Fat Diet Mice
Source: PLoS One. 2015 Sep 23;10(9):e0138832. doi: 10.1371/journal.pone.0138832 (PMC4580448; doi:10.1371/journal.pone.0138832)

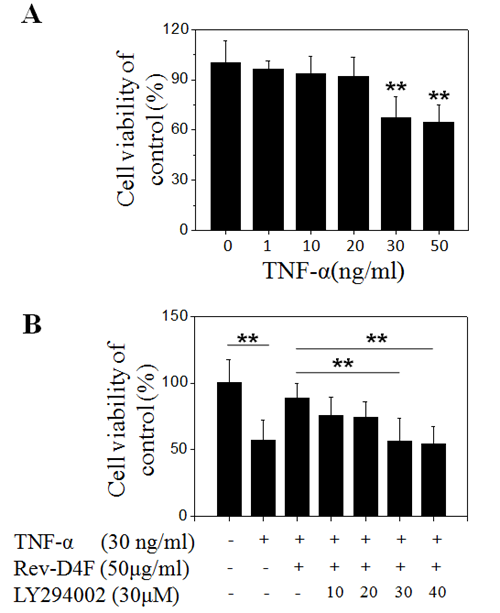

Supplement: S1 Fig — A, The effect of different concentration of TNF-α on EPCs viability. **P <0.01 versus TNF-α (0 ng/ml). B, LY294002 inhibited the effect of Rev-D4F on EPCs viability. Samples were pretreated with different concentrations of a PI3K inhibitor LY294002 for 2 h and incubated with Rev-D4F (50μg/ml) for 6h, EPCs were treated with TNF-α for 24h to detect the functions of viability. **P <0.01. (TIF) [file pone.0138832.s001.tif]

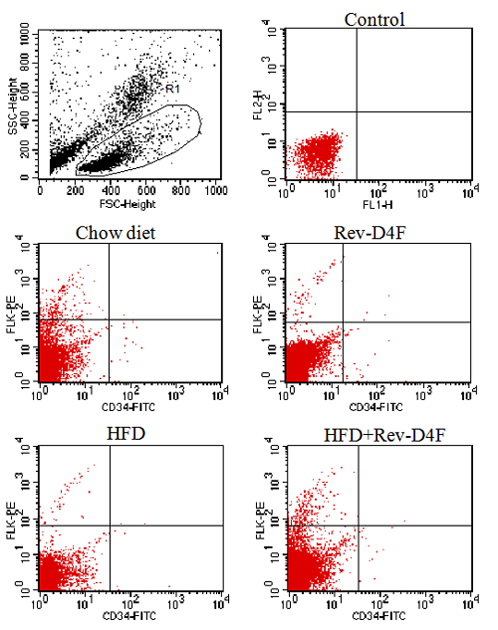

Supplement: S2 Fig — Quantification of CD34+ and CD34+FLK-1+ were shown in S2 Fig. Control represents isotype-identical antibodies. (TIF) [file pone.0138832.s002.tif]

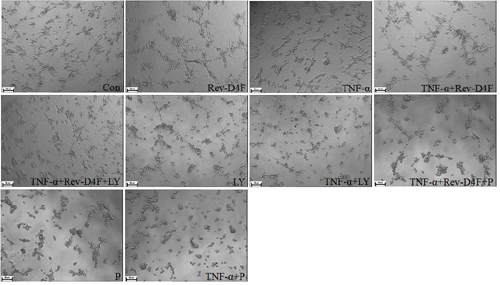

Supplement: S3 Fig — legend. Cells were pretreated with a PI3K inhibitor LY294002 (30μM) or AKT inhibitor perifosine (5μM) for 2 h and incubated with Rev-D4F (50μg/ml) for 6h, EPCs were treated with TNF-α for 24h to detect tube formation. Scale bar represented 50μm. (TIF) [file pone.0138832.s003.tif]
